# St. Louis Hospital

**Published:** 1830-04-01

**Authors:** 


### CLINICAL REVIEW.

### XXXIX.

#### HOPITAL SAINT LOUIS.

#### I. Cæsarian Operation on a Patient dead from Hæmoptysis.*

The following is a curious instance of the Cæsarian operation saving the life of the child some minutes after the death of the mother. We question much whether a similar step would have been resorted to in this country, save and except by those few choice spirits who possess a true German relish for gastrotomy.

*Case.* Eliza Doviller, æt. 28, entered the Hôpital St. Louis on the 2d of June, 1829, with the usual symptoms of phthisis pulmonalis; cough, blood streaked expectoration, sharp pains in the chest, hectic fever, emaciation. The resonance of the chest was increased below the clavicles, a circumstance due, as the intelligent reporter remarks, partly to the emaciation of the subject and partly to tuberculous excavations; the hand applied in these regions had a *fremissement* communicated to it by the voice; the natural respiration gave way in these situations to *gargouillement* and pectoriloquy; and finally the breathing elsewhere was more sonorous than usual; auscultic symptoms that indicated, but too unerringly, the mischief in the pulmonary texture. Besides this mortal disease the unfortunate patient was eight months and a half gone in pregnancy.

On investigating her history it appeared that she had always led a very irregular life; that four years before

* Journal Hebdomadaire. No. 59.

her admission she was seized with bronchitis, which in spite of the measures had recourse to passed into a chronic state; that continuing her excesses the cough grew daily worse, and became accompanied with pains in the chest, expectoration, and emaciation; and that finally, in spite of the earnest advice of her physician she left her situation of femme-de-chambre to live with her paramour. Soon after this step the menses ceased to flow, and the usual symptoms of pregnancy appeared. At first this change gave a marked relief to the thoracic complaint, but as soon as the uterus acquired any magnitude the pains in the chest and suffocation returned, she began to spit blood, and her weakness rapidly increased.

We need not particularize the treatment pursued in the hospital, suffice it to say that on the 14th, the weather being hot and oppressive at the time, she suffered much from suffocation and spat more blood than usual. A small bleeding was had recourse to and procured some relief, but at 7 P. M. of the 17th she was suddenly seized with a frightful hæmoptysis, and expired in the space of four minutes, with the blood gushing from the mouth and nostrils.

When the interne, M. Huguier, arrived, life was quite extinct, and he instantly determined to lay open the uterus and save the life of the child if possible. The corpse being placed with the head and chest raised by pillows and the thighs flexed upon the pelvis, an assistant retained the uterus in the middle of the abdomen. The integuments then being put upon the stretch by the left hand applied below the umbilicus, a vertical incision was made with a convex bistoury along the median line, commencing at an inch below the umbilicus and terminating at an inch and a half or two inches from the pubes. The skin, cellular tissue, linea alba, and peritoneum were all cut through, with a vast deal of superfluous caution considering that the patient was a dead one, and at length the anterior and superior portion of the uterus was incised, "layer by layer," till the membranes were exposed, punctured, and the liquor amnii evacuated. The fore and middle fingers of the left hand were introduced into the opening and the latter enlarged by tearing the membranes, when the whole hand was got into the cavity of the womb, and the sides of the incision separated, by the fingers being held wide apart at its superior angle.

The feet of the child were now sought for with the right hand and quickly found, when both hands were withdrawn and employed to extract the infant which scarcely gave any signs of life. It was pale, exsanguined, motionless, and the pulsations of the heart were scarcely perceptible. The cord was tied before it was cut, frictions with warm cloths on the præcordia were had recourse to, artificial respiration set up, and the child placed in the warm bath. Under the employment of these measures the heart began to beat more vigorously, the nisus of respiration was induced, and the infant uttered that feeble cry which heralds our entrance into a stormy existence. The animal heat could only be retained by keeping the little creature in warm cloths, but at the age of thirty days, when the report closes, it enjoyed good health.

No dissection of the unfortunate mother was permitted by the friends, but that does not matter very much, as the rescue of the offspring by the Cæsarian section is the important portion of the case. Whether an existence so procured will be one of happiness or misery to the infant, whether it bears in its organization the germs of its future destruction by scrofula or phthisis, or whether it does not, it must at least be allowed that no case can possibly arise, in which the Cæsarian operation can inflict a less amount of suffering, in proportion to its good, than it has done in the present instance. The knife of the surgeon or the anatomist "wrings not the tethers" of the dead!

II. SEVERE SYMPTOMS AFTER OPENING A BODY.*

The interne of this hospital, M. T—,

* Journ. Hebd. No. 56.

examined the body of a woman who died of puerperal fever, having at the time a small abrasion on the extremity of one of his fingers. On the day of the dissection, about noon, he felt a very severe pain in the part, which was red and swollen, and in two hours afterwards the glands in the axilla swelled and grew painful likewise; at the same time several red and tortuous lines appeared on the back of the hand and fore-arm. M. T. now began to experience malaise, head-ache, a sense of heat in the throat, and some shiverings. He went out in order to drive away his morbid feelings, but suffered from pains and aches in his limbs and extreme fatigue. At 6, p. m. he experienced thirst with a sensation of burning in the bronchia, œsophagus, and epigastrium; the mouth was dry and clammy. He took some soup and eat a few grapes, shortly after which he was attacked with a violent shivering that lasted three hours, and was accompanied by nausea and syncope. M. T— went to bed and slept for some hours, but in the middle of the night he was seized with fever, agitation, and delirium. At 4, a.m. he sweated profusely and again went to sleep. In the morning a small vesicle was observed at the excoriated part of the finger, at the bottom of which was a little slough, and around it was a tense areola. The red streaks along the hand and fore-arm were no longer seen, but the axillary glands continued swollen, and the pain extended throughout that side of the chest. The general symptoms, which were not so severe, consisted in—head-ache, oppression at the epigastrium and general malaise, pale anxious countenance, small and feeble pulse, foul clammy tongue. The sore was freely cauterised with the nitrate of silver, and some negus of old wine, rendered aromatic by the admixture of canella, was taken alternately with strong tea throughout the day. Two hours after the commencement of this plan the head-ache and nausea disappeared, a profuse perspiration broke out and continued throughout the day, and in the evening the patient was nearly well. On the third day very little swelling and pain in the axilla remained, the slough made by the nitrate of silver soon separated, and thus M. T— got thoroughly out of a scrape that has proved fatal to too many professional men in Europe and America.

We cannot part from this interesting topic, interesting at least amongst ourselves, though we fancy that small sympathy is bestowed upon us by the public, without giving what we believe to be an useful caution. From what we have seen we should say that cauterization of these pricks in dissection, whether by the nitric acid, the nitrate of silver, the caustic potass, or any other method, is productive of mischief. Irritation and inflammation are produced if the habit of the individual be indifferent, which it commonly is; a troublesome ill-conditioned sore ensues; and weeks, months, nay even years may elapse before this apparently trifling puncture is fairly healed. Of this we have seen many instances amongst students, and we have before our eyes the case of a young man who applied the nitric acid to a prick on the back of his hand, and suffered for nine months from as foul and irritable a looking sore as one often sees. We should say that in the milder forms of constitutional implication from dissection-wounds, the best plan is to use only poultices or soothing applications to the part, take a brisk purge or two, and support the system by such tonics as a fair allowance of port wine or malt liquor, according to circumstances, with a liberal diet. These means succeeded by a little country air, are far more efficacious in ordinary cases than any others with which we are acquainted. The treatment of those acute and frightfully fatal cases that now and then occur is quite a different matter, a subject in fact on which we have no intention of entering at present.

### III. Remarks by Baron Alibert on the Leprous Affection termed Leuca by the Ancients.*

M. Alibert observes that this affection, *Leuce* or *Leuca*, is very rare in the present age, and appears to have been

* Revue Medicale. Sept. 1829.

replaced by elephantiasis. M.M. Quoy and Gaimard in their voyage round the world have noticed it in the natives of several countries, and M. Alibert himself has seen a few examples of it. We shall lay before our readers a concise description of the disease, which boasts of more ancient mention in historical documents than any other cutaneous complaint.

It begins by spots, *taches*, of a wholly unusual appearance; they are first of a whitish or ash grey colour, sometimes of a greenish white with a shade of yellow. Though occasionally irregular they are most commonly circular, bordered with an inflamed areola of reddish or rosy tint. These characters are pretty constant in the first stage of the disease, which still preserves the level of the healthy integuments. In the *second* stage, the patches become larger, more brown or black, grow hard, and are depressed; the areola remains but the diseased skin is deprived of its sensibility. In the *third* stage, the patches become of a very firm consistence, almost coriaceous, and the areola disappears. The depression of the skin in the second stage is a specific character and is dwelt on in Leviticus. M. Alibert was consulted by a planter of Louisiana, in whom this complaint had developed itself almost insensibly on the left side of the abdomen. The dry and discoloured integument was marked by white, circular spots, each becoming scaly in its turn by the progressive desiccation of the cuticle. These patches originally of a greyish white hue lost this colour in the course of some months after their appearance, when they grew browner, wrinkled in the centre, and at last exhibited a very visible depression below the level of the surrounding surface. The disease increased rapidly and could not be checked. The eruption is accompanied with scarcely any itching, but is generally preceded by a feeling of weakness in the system, and a kind of languor in the exercise of the functions of the body. When once established, the characters previously enumerated will amply suffice to distinguish this terrible disease, which excited such horror in the ancient world, that both in Asia and Europe lepers were legally dead, and we read that Tamerlane the ferocious Tartar conqueror ordered their utter extermination in all the countries which submitted to his arms. M. Alibert enters with much eloquence into the *history* of this variety of leprosy, but as such a disquisition is rather a matter of curiosity than practical interest we shall waive accompanying the learned Baron. As he makes no mention of the treatment we suppose he knows of none possessed of any efficacy.

